# Development and characterization of microsatellite markers for diploid populations of the wind-pollinated herb *Mercurialis annua*

**DOI:** 10.1186/s13104-017-2700-z

**Published:** 2017-08-10

**Authors:** Ana Paula Machado, John R. Pannell, Jeanne Tonnabel

**Affiliations:** Department of Ecology and Evolution, Biophore Building, University of Lausanne, 1015 Lausanne, Switzerland

**Keywords:** Population genetics, Phylogeography, Metapopulation dynamics, Microsatellites, Range expansion, Sexual systems, Dioecy

## Abstract

**Objective:**

*Mercurialis annua* is a wind-pollinated annual plant that has long been used as a model for the study of ploidy and sexual-systems evolution. However, no molecular markers are yet available for genetic studies of its diploid populations. Here, we develop and characterize a set of eight polymorphic microsatellite markers for diploid dioecious *M. annua*.

**Results:**

Following an SSR-enrichment protocol, 13 microsatellite markers were proposed, eight of which yielded successful amplification and polymorphism. We screened the eight microsatellite loci in 100 individuals. The number of alleles per marker ranged from 6 to 12, and observed heterozygosity ranged from 0.57 to 0.76. To estimate potential allele scoring errors, these individuals’ offspring were genotyped for the same loci, and error rates were estimated from parentage analyses. Error rates ranged from 0 to 6.8%. Cross-amplification tests were performed for congeneric *M. huetti* and *M. canariensis*, with successful amplification for seven and six of the eight loci, respectively. The novel microsatellite markers proposed here will be crucial for a multitude of genetic studies of *M. annua* and further establish its importance as a model species for addressing ecological and population genetic questions.

## Introduction

*Mercurialis annua* is a wind-pollinated annual species that can be found in central and western Europe and around the Mediterranean Basin [1]. Across its distribution, *M. annua* displays remarkable variation in both ploidy, ranging from diploid to 12-ploid, and sexual system, ranging from dioecy to monoecy through androdioecy [1]. The species was first used as a model to address sex determination questions in the late 19th and early 20th centuries [2, 3]. In the 1960s, it became the focus of study of the evolution of ploidy and sexual systems [1]. More recently, *M. annua* has been used in studies of population range-expansion [e.g. 4] a topic that naturally requires the use of modern molecular genetic tools. For more information on the value of *M. annua* as a model system see [5] and references therein.

The scope for population-genetic and mating-system studies in diploid *M. annua* is currently limited by a lack of modern genetic markers. Indeed, the tools available are limited to isozyme loci [6], internal transcribed space (ITS) and chloroplast DNA sequences [7]. Although microsatellite markers have been successfully developed for the allohexaploid *M. annua*, these do not amplify in the diploid form [8]. Due to their high mutation rate and high polymorphism, microsatellites are powerful markers to detect genetic variation, allowing us to distinguish between closely related individuals, and thus to assess parentage and kinship relationships [9]. Additionally, it is a cost-effective PCR-based technique that does not require large quantities of DNA. As these qualities render microsatellites extremely advantageous genetic markers, we aimed to design a set of such markers that we could use in future work on diploid *M. annua*. Particularly, we are interested in studying selection gradients and kin selection in addition to metapopulation processes and population subdivision. Hence, we developed eight polymorphic microsatellite markers for diploid dioecious *M. annua* and examined cross-species amplification success in two other *Mercurialis* species.

## Main text

Microsatellite markers were developed from DNA extractions of 15 individuals following an enrichment protocol at ecogenics GmbH (Balgach, Switzerland). Size-selected fragments from genomic DNA were enriched for simple sequence repeat (SSR) content using magnetic streptavidin beads and biotin-labelled CT and GT repeat oligonucleotides. The SSR-enriched library was analysed on an Illumina MiSeq platform using the Nano 2 × 250 v2 format. After assembly, 756 contigs/singlets contained a microsatellite insert with a tetra- or a trinucleotide of at least 6 repeat units or a dinucleotide of at least 10 repeat units. Of these, 465 contigs were suitable for primer design, pointing to the potential of 13 microsatellites markers with number of alleles ranging from 3 to 8. Individual tests for amplification and polymorphism were performed on 15 individuals, after which eight of the 13 potential markers were retained (Table 1). Markers of similar annealing temperature (T_a_) with different fluorescent tags and size ranges (Table 1) were grouped in three multiplexes. Primer3 [10] was used to avoid primer hybridisation within the multiplexes.

**Table 1:** Characteristics of the three primer multiplexes developed for *M. annua*, tested on 100 individuals

M	Locus	Primer sequences (5′–3′)		Repeat motif	Size range (bp)	Ta (°C)	FC (µM)	N_A_	*H*_O_	*H*_E_	Error rate %
1	1101	F	*5′FAM*—ACTAAAGTCCACTCATGGATGC	(TCTT)_9_	94–115	60	0.1	6	0.580	0.599	1.8
		R	GTATCCGTCCCTGGAGGAAG								
	661	F	*JOE*—CAACGCTTTTGTACAGGGGG	(CTAT)_7_	187–203	60	0.1	6	0.620	0.685	0.0
		R	ACATTTATCCCTCCCCAGGC								
	7575	F	*AT550*—AGTCACAGCTTGCAAATCTCC	(TACA)_7_	222–255	60	0.1	12	0.690	0.851	6.8
		R	TGCATCTTCTGGTTTCATCCC								
2	317	F	*5′FAM*—TTTACCGAGATGCTCCCCAG	(CTAT)_7_	188–273	60	0.2	11	0.760	0.804	1.9
		R	AAAGCCCCTGGAATTGTTGC								
	13654	F	*JOE*—TGCTGTTGGGATAAACAGGC	(TCTT)_9_	191–219	60	0.2	7	0.730	0.792	1.1
		R	CACTGCAAACGCATGAATCG								
	4048	F	*AT550*—AGGGGTACCGTTTTGAGTCC	(GT)_12_	212–240	60	0.2	8	0.570	0.778	5.9
		R	CGATCCCTGCACAATCTGAC								
3	330	F	*HEX—*TAGCACGGAAACAAAAACGC	(AAAG)_8_	168–196	59	0.2	9	0.670	0.769	0.8
		R	CTCGGTGGATTACCAAGACG								
	3889	F	*5′FAM*—TCGGTTTGATTTTTGAACACCC	(TCTA)_7_	216–242	59	0.2	7	0.710	0.696	0.0
		R	CACTGTAATCATGCCAAAGCTG								

Fluorescent tags attached to the 5′ end of the forward primers and are indicated in italic

M, multiplex; Ta, annealing temperature; FC, final concentration of each set of primers in the PCR reaction; N_A_, number of alleles; *H*_*O*_, observed heterozygosity; *H*_E_, expected heterozygosity

To characterize variation at the eight chosen loci, we genotyped 50 males and 50 females that had been sown as part of an outdoor common garden experiment at the field platform of the LabEx CeMEB in Montpellier (France). These plants originated from a mix of European populations encompassing the natural distribution of diploid *M. annua*. Total DNA was extracted using the BioSprint 96 DNA Plant Kit (Qiagen, Germany) following the manufacturer’s instructions, and eluted in 100 µl of distilled water. PCR amplification was carried out in a final volume of 10 µl, including 2× Multiplex PCR Master Mix (Qiagen, Germany), 1 µl of extracted DNA, and variable primer concentrations (Table 1). Thermal cycling was performed in a TProfessional Standard Thermocycler (Biometra GmbH, Göttingen, Germany) as follows: 95 °C for 15 min; 35 cycles of 94 °C for 30 s, Ta (annealing temperature of each multiplex) for 90 s, 72 °C for 90 s; and a final step at 72 °C for 10 min. Diluted PCR products (1/100) were analysed by capillary electrophoresis on an ABI3100 Genetic Analyzer (Applied Biosystems), with internal size standard GeneScan-350 LIZ. Fragment length analyses and scoring were performed with GeneMapper v4.0 (Applied Biosystems). Arlequin v3.5.2.2 [11] was used to calculate genetic diversity parameters, assess pairwise linkage disequilibrium (LD) between loci and test for Hardy–Weinberg (HW) equilibrium.

To estimate the accuracy of allele scoring, 7–8 seeds per female (total of 391 seeds) were sown at the greenhouses of the Department of Ecology and Evolution of the University of Lausanne, Switzerland, and the resulting plants were genotyped for all markers. For each marker separately, we counted a mismatch when the offspring and its mother had no alleles in common. The error rate per marker was calculated as the proportion of mismatches across all offspring.

The number of alleles per locus ranged from 6 to 12, the observed heterozygosity ranged from 0.57 to 0.76, and the expected heterozygosity ranged from 0.60 to 0.85 (Table 1). Several markers showed significant linkage disequilibrium (LD) and deviations from Hardy–Weinberg (HW) equilibrium. Given that the tested samples were the offspring of the third generation of crosses within a small population, HW deviations are to be expected. For the same reason, the signal for LD is likely to reflect high relatedness between the individuals rather than physical linkage between the markers. Allelic frequencies across all markers ranged from 1 to 52.5%. The error rate per marker ranged from 0 to 6.8% (Table 1).

Cross-amplification was checked in two sister species of *M. annua*: *M. huetii* (diploid) and *M. canariensis* (tetraploid). Five individuals of each sister species were genotyped for the three multiplexes in the same conditions as those described above, and similar genetic diversity calculations and tests for HW equilibrium were also performed. For the tetraploid *M. canariensis*, microsatellite scoring of alleles followed the gene dosage method, as described in [12], and genetic diversity parameters were calculated with AUTOTET [13].

The amplification in sister species was successful for most markers in both species, with the exception of locus 4048, which produced unspecific amplification and was therefore removed from the analyses, and locus 7575, which did not amplify in *M. canariensis* (Table 2). Loci 13654 and 661 were monomorphic for *M. canariensis* and *M. huetii*, respectively.

**Table 2:** Cross-amplification of microsatellite markers in *M. annua* sister species

Locus	*M. canariensis* (4n)				*M. huetii* (2n)			
	N_A_	Size Range(bp)	*H*_O_	*H*_E_	N_A_	Size range (bp)	*H*_O_	*H*_E_
1101	3	94–111	0.467	0.453	3	94–115	1.000	0.644
661	6	209–325	0.567	0.742*	1	187	–	–
7575	0	–	–	–	5	226–255	0.200	0.867*
317	8	196–216	0.933	0.784	3	169–197	1.000	0.689
13654	1	184	–	–	3	184–215	0.000	0.622*
330	4	172–187	0.567	0.583*	4	172–191	0.800	0.733
3889	3	230–242	0.500	0.471	3	216–242	0.600	0.689

Locus 4048 is not shown as it did not amplify in any of the species. No significant linkage disequilibrium (LD) was found between loci

* Significant deviation from HW equilibrium (*p* value threshold at 0.0071 with Bonferroni correction)

To our knowledge, no microsatellite loci have been developed for diploid *M. annua* to date (but note the existence of primers designed for allohexaploid *M. annua* [8]). The set of loci proposed here will be useful for further research in *M. annua*. In particular, studies of its range expansion, spatial population genetics, mating systems and paternity assignment will be greatly facilitated by these markers. The low error rates and the presence of rare alleles render them particularly useful for paternity analyses, which is of prime importance to understand several aspects of the evolution of sexual systems, including sexual selection, the evolution of sexual dimorphism and the estimation of fitness gain curves. Since most markers also amplified in the two congeneric species tested, our study broadens the perspectives of studies for these species too, as well as studies of homoploid and polyploid hybridization.

## Limitations

- For some studies, eight markers may not be sufficient, for example to detect weak levels of structure within and between populations.
- The use of individuals from our common garden experiment after a few generations of crosses within a relatively small population may have yielded lower levels of polymorphism per marker than a wild population would.

## Abbreviations

SSR: simple sequence repeats
Ta: annealing temperature
PCR: polymerase chain reaction
LD: linkage disequilibrium
HW: Hardy–Weinberg
